# Comparison of Feed Digestibility between Ponies, Standardbreds and Andalusian Horses Fed Three Different Diets

**DOI:** 10.3390/vetsci9010015

**Published:** 2021-12-31

**Authors:** Samantha J. Potter, Nicholas J. Bamford, Courtnay L. Baskerville, Patricia A. Harris, Simon R. Bailey

**Affiliations:** 1 Faculty of Veterinary and Agricultural Sciences, The University of Melbourne, Parkville, VIC 3052, Australia; pots@student.unimelb.edu.au (S.J.P.); basc@student.unimelb.edu.au (C.L.B.); bais@unimelb.edu.au (S.R.B.); 2 Waltham Petcare Science Institute, Melton Mowbray LE14 4RT, Leicestershire, UK; pat.harris@effem.com

**Keywords:** digestibility, equine, insulin dysregulation, laminitis, nutrition, obesity

## Abstract

Ponies and some horse breeds such as Andalusians exhibit an ‘easy keeper’ phenotype and tend to become obese more readily than other breeds such as Standardbreds. Various hypotheses have been proposed, including differences in appetite or metabolic efficiency. This study aimed to investigate the effect of breed on nutrient digestibility. Ponies, Standardbreds and Andalusian horses were adapted to consuming either a control fibre-based diet (*n* = 9), a hypercaloric cereal-rich diet (*n* = 12) or a hypercaloric fat-rich diet (*n* = 12) over 20 weeks. Total faecal collection was performed over 24 h to determine apparent total tract digestibility of gross energy, dry matter (DM), neutral detergent fibre (NDF), starch, crude protein and crude fat. There was no effect of breed on apparent digestibility for any of the nutrients studied (all *p* > 0.05). However, there was a significant effect of diet, with animals consuming the cereal-rich or fat-rich diets demonstrating higher digestibility of gross energy, DM, NDF and crude protein compared with those consuming the control diet (all *p* < 0.05). Animals adapted to the cereal-rich diet demonstrated higher digestibility of starch (*p* < 0.001) and animals adapted to the fat-rich diet demonstrated higher digestibility of fat (*p* < 0.001). This study found that horses and ponies had similar nutrient digestibility when adapted to the same diets and management conditions. Limitations included the relatively small number of animals from each breed per diet group and the short period of total faecal collection. The tendency towards increased adiposity in ponies and Andalusian-type horse breeds is more likely to reflect differences in metabolism, rather than differences in feed digestibility.

## 1. Introduction

Obesity is an increasing concern among equine populations due to its potential association with a number of serious health conditions including insulin dysregulation and laminitis [1]. Ponies and several horse breeds tend to gain weight easily, often developing regional adiposity and a characteristic ‘cresty neck’ appearance, known colloquially as ‘good doers’ or ‘easy keepers’ [2]. A likely explanation for increased adiposity in these breeds is the observation of increased insulin responses to oral non-structural carbohydrates (NSC), even when in moderate body condition, as insulin is known to promote fat deposition and inhibit lipolysis [3]. However, it is also possible that rates of weight gain and changes in body condition may be attributed to differences in metabolic efficiency or nutrient digestibility between breeds.

Nutrient digestibility has been compared between ponies and horses in several studies with conflicting results. One study reported a tendency toward higher digestibility of dry matter (DM), crude fibre (CF) and neutral detergent fibre (NDF) in ponies compared with horses [4], while another also found that ponies had a tendency toward higher digestibility of CF [5]. Higher digestive efficiency in ponies might seem logical in the evolutionary context of pony breeds that adapted to thrive on relatively sparse low-quality forage with high fibre content. However, other studies have been unable to support these results and did not detect differences in nutrient digestibility between ponies and horses [6,7,8,9]. Recently, higher digestibility of CF, dietary fibre and starch was found in Icelandic horses compared with Danish warmbloods [10], suggesting that further work is needed.

The present study aimed to compare feed digestibility in three different equine breeds: two horse breeds of similar size with one breed recognised to be relatively insulin sensitive (Standardbreds) and one breed recognised to be relatively insulin resistant (Andalusians), and a group of ponies. Additionally, animals were adapted to consuming one of three different diets: a control fibre-based diet, a hypercaloric cereal-rich diet or a hypercaloric fat-rich (low-NSC) diet. Our hypothesis was that ponies and Andalusian horses would have higher apparent digestibility of nutrients compared to Standardbred horses when adapted to the same diets.

## 2. Materials and Methods

### 2.1. Study Design and Diets

The digestibility study occurred immediately following the conclusion of a longitudinal diet study in which the metabolic profiles of horses and ponies, including morphometrics, insulin sensitivity and adipokine concentrations, were characterised and reported elsewhere [11]. These studies were approved by the University of Melbourne Animal Ethics Committee (approval number 1011918.5). Briefly, 11 Standardbred horses (9.5 ± 1.8 years; 8 geldings and 3 mares), 11 mixed-breed ponies (9.0 ± 1.2 years; 7 geldings, 4 mares) and 11 Andalusian horses (8.3 ± 1.2 years; 3 geldings and 8 mares) were adapted over 20 weeks to consuming one of three different study diets. Each diet group comprised an equal number of each breed (control diet group, *n* = 3 per breed totalling 9 animals; cereal-rich and fat-rich diet groups, *n* = 4 per breed totalling 12 animals per diet group). Animals were blocked by breed and randomly allocated to one of the three diet groups using a random number generator. Using a standard deviation value of 5 and *p* value (α) of 0.05, the number of animals in each breed group was estimated to be able to detect a difference in apparent digestibility of 10 percentage points with a power of 80%.

Horses and ponies were kept in small groups in dry lot paddocks with ad libitum access to fresh water and a low-NSC mixed grass hay for the duration of the study. They were brought into individual side-by-side pens twice daily (0800 and 1600 h) to receive complementary meals, which differed depending on the diet group.

The first diet group received a control fibre-based ration consisting of oaten chaff, soaked soya bean hull pellets (Maxisoy, Energreen Nutrition), plus a powdered vitamin and mineral supplement (Ranvet), the amount of which was adjusted to body weight to meet estimated maintenance energy requirements [12]. The second diet group received a cereal-rich ration consisting of micronised maize (Micrmaize, Hygain), oaten chaff, soaked soya hull pellets (Maxisoy) plus a vitamin and mineral supplement (Ranvet), which was adjusted to body weight to exceed maintenance requirements and promote weight gain. The third diet group received a fat-rich ration consisting of granulated vegetable fat (Cool Calories, Buckeye Nutrition) and canola oil (Energy Gold, Kohnke’s Own), lucerne chaff, soaked soya hull pellets (Maxisoy) plus a vitamin and mineral supplement (Ranvet), which was also adjusted to body weight to exceed maintenance requirements and promote weight gain. To avoid digestive disturbances, the amounts of micronised maize in the cereal-rich group or vegetable fat/oil in the fat-rich group were gradually increased over the study period, reaching levels that provided approximately 200% of daily digestible energy requirements [12]. The composition of the study diets at the time of the digestibility study is shown in Table 1, with the proximate analysis performed at Equi-Analytical (Ithaca, NY, USA).

The characteristics of horses and ponies at the time of the digestibility study are shown in Table 2. Pony breeds included Connemara pony (4), Australian pony (3), Welsh pony (3) and unknown pony breed (1). Body condition score (BCS) and cresty neck score (CNS) were evaluated by a single experienced investigator using a 9-point scale [13,14] and 5-point scale [15], respectively, and body weight was measured using calibrated weigh scales. Animals in the cereal-rich and fat-rich diet groups reached ‘obese’ body condition after 20 weeks, while animals in the control group had maintained ‘moderate’ body condition [11].

### 2.2. Sample Collection and Analysis

For the digestibility study, animals were kept in individual pens for a 24-h period to facilitate the measurement of total feed intake and collection of total faecal outputs. The ground surface of the pens was covered with clean and dry wood chips to reduce contamination and enable complete removal of faeces. There was no precipitation during the collection period and ambient temperature ranged from 14 to 22 °C. Animals were provided with ad libitum access to fresh water and the same batch of low-NSC mixed grass hay that they received during the diet study. Hay was weighed prior to placing in feeders and any refusals were collected and weighed to calculate total hay intake. The total amount of faeces per animal was collected at 1000, 1300, 1600, 1900, 2200, 0500 and 0800 h. Faeces were separated from any adherent wood chips and immediately weighed, prior to placing in large plastic bags. Once the 24-h collection period was completed, faeces were thoroughly agitated within the large plastic bags prior to collection of 300–400 g composite samples that were placed in clean foil containers. Samples of hay and each of the three complimentary feeds were similarly collected and placed into clean foil containers. All samples were weighed and sealed before storing at −20 °C pending analysis.

Samples of feeds and faeces were sent to a commercial laboratory (SGS Food & Agricultural Laboratory, Toowoomba, QLD, Australia) where analyses were performed according to the methods of the Association of Official Analytical Chemists (AOAC) International. These included gross energy (bomb calorimetry; method reference AS1038), DM (two stage moisture calculation; method reference MST001), NDF (sequential extraction method), starch (total; method reference STA-001), crude protein (Dumas combustion; method reference PRN002) and crude fat (solvent extraction; method reference OIL001).

### 2.3. Data Analysis

Percentage digestibility for each nutrient was calculated and compared between groups. Apparent total tract digestibility of nutrients was calculated as:

Digestibility (%) = [(intake (g) − faecal excretion (g)) ÷ intake (g)] × 100

Statistical analysis was performed using the general linear model function of SPSS (version 23; IBM Corporation, New York, NY, USA) and graphics were created using GraphPad Prism (version 9.2; GraphPad, San Diego, CA, USA). For each dependent variable, breed, diet, and the interaction of breed × diet were included as fixed effects. Simple main effects were compared using Bonferroni’s post hoc test and assumptions of the model were checked using the Shapiro–Wilk test (normality of residual values) and Levene’s test (homogeneity of variance). Significance was accepted at *p* < 0.05.

## 3. Results

### 3.1. Feed Intake and Faecal Output

There were no adverse events or meal refusals during the 24-h collection period. Hay intake was not different between diet groups; however, total DM intake was higher in horses and ponies consuming the cereal-rich diet, owing to higher DM content of the cereal-rich complimentary meals (Table 3). There were no differences in faecal moisture content (DM percentage) or total faecal DM output between diet groups. There were no significant effects of breed on any of the feed intake or manure output variables evaluated (all *p* > 0.05).

### 3.2. Apparent Total Tract Digestibility

**Figure 1:**
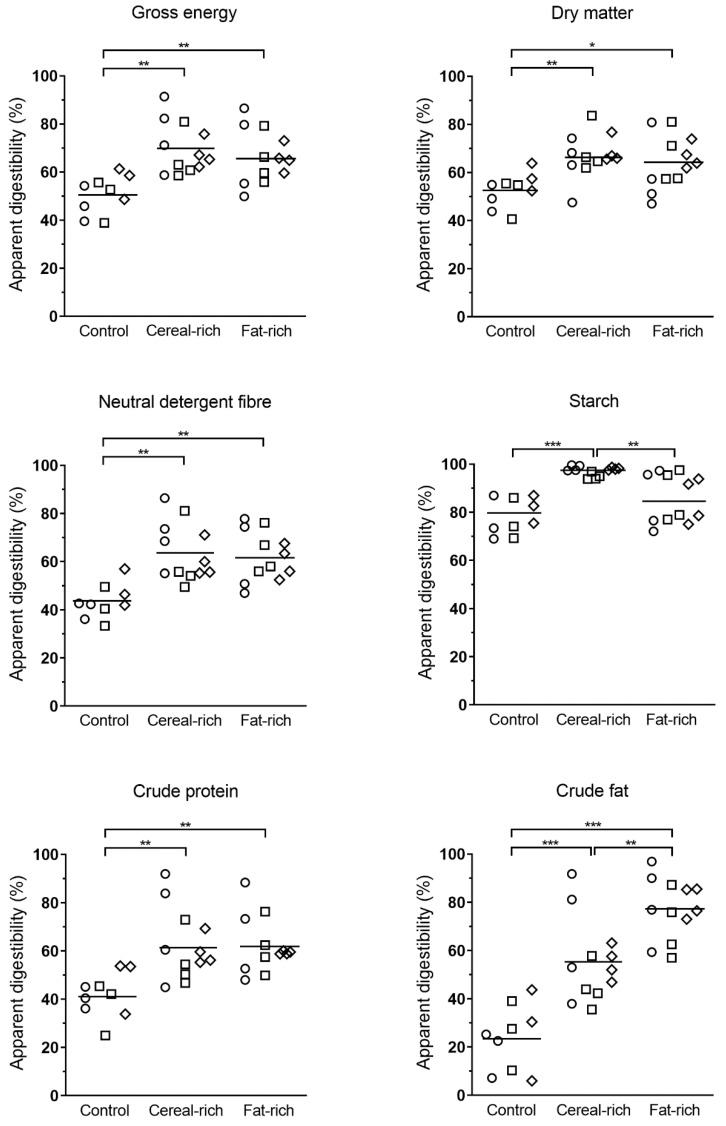
Apparent total tract digestibility of nutrients in Standardbred horses (circles), ponies (squares) and Andalusian horses (diamonds) adapted to eating a fibre-based control diet, hypercaloric cereal-rich diet or hypercaloric fat-rich diet over 20 weeks. No effect of breed was detected for any nutrient (all *p* > 0.05). Horizontal lines indicate diet group means. Pairwise comparisons performed between diet groups with Bonferroni correction (* *p* < 0.05, ** *p* < 0.01, *** *p* < 0.001).

There were no significant effects of breed on apparent digestibility of any the nutrients evaluated (all *p* > 0.05). However, there was a significant effect of diet for all variables analysed (Figure 1; Supplementary Table S1). Horses and ponies consuming both the cereal-rich and fat-rich diets demonstrated higher digestibility of gross energy, DM, NDF and crude protein compared with those consuming the control diet (all *p* < 0.05). There was a significant effect of diet on starch digestibility, with animals adapted to the cereal-rich diet demonstrating higher digestibility of starch than those consuming the fat-rich (*p* = 0.005) or control (*p* = 0.001) diets. There was also a significant effect of diet on the apparent digestibility of fat, with animals adapted to the fat-rich diet demonstrating higher digestibility of fat compared with those consuming the cereal-rich (*p* = 0.005) or control (*p* < 0.001) diets. Interestingly, animals adapted to the cereal-rich diet also showed greater fat digestibility compared with those consuming the control diet (*p* = 0.001).

## 4. Discussion

This study did not detect an effect of breed on nutrient digestibility, thereby failing to support our hypothesis that ponies and Andalusian horses would have higher digestibility of nutrients compared to Standardbred horses when adapted to the same diets. Limitations of this study included the relatively small number of animals from each breed per diet group and the short period of total faecal collection. The possibility of differences in nutrient digestibility between equine breeds in general cannot be discounted on the basis of our findings. However, the animals studied were well characterised regarding their metabolic traits, which did show clear differences between breeds, despite the absence of detectable differences in apparent digestibility.

There is interest in comparing the breeds of ponies and horses that typically present with obesity and insulin dysregulation with reference breeds such as Thoroughbreds and Standardbreds, to understand the physiology that underpins differences in metabolic phenotype [2]. Andalusian horses were used in this study as a horse breed that typifies the tendency to exhibit a ‘cresty neck’ and regional or generalised obesity, particularly when given access to lush grass or grain-based diets, and being predisposed to laminitis [16]. The metabolic characteristics of the same animals studied here have previously been reported [11], in which differences between breeds were consistent across diet groups, with ponies and Andalusian horses demonstrating lower insulin sensitivity and higher acute insulin responses to glucose compared with Standardbred horses. Further, there was an effect of diet observed, with animals in the cereal-rich diet group demonstrating overall lower insulin sensitivity and higher acute insulin responses to glucose compared with the control and fat-rich diet groups, which were similar to each other.

Differences in apparent total tract digestibility were demonstrated between groups adapted to consuming different diets. This study occurred following a longitudinal metabolism study that lasted 20 weeks, during which time animals were introduced gradually to relatively large amounts of cereal grain or fat/oil in their diets. No animals showed any apparent negative side effects of either hypercaloric ration, potentially due to this slow acclimation period. Animals in the cereal-rich and fat-rich diet groups demonstrated higher body condition and cresty neck score compared to the control group, having developed obesity at the conclusion of the metabolism study. This meant that our study was unable to separate the effects of diet and obesity on apparent digestibility. It might have been informative to evaluate nutrient digestibility on several occasions during the feeding period, especially when all animals were in moderate body condition, although they also would not have been as well adapted to the study diets. Two previous studies failed to detect an effect of obesity on the apparent digestibility of dietary gross energy or DM when comparing lean and obese ponies [17] or lean and obese horses [18]. Therefore, it is unlikely that obesity itself would contribute to differences in feed digestibility, although the potential effect of adiposity (at different levels) on nutrient digestibility has not been widely investigated in equids and this aspect could warrant further investigation.

The equine digestive tract, remarkably, seems to adapt very well to diets that contain large amounts of fat/oil. The activity of pancreatic lipase has been shown to be similar between adult horses, pigs, and rats [19]. It appears that in most equine diets with added fat/oil, the apparent digestibility for the added fat may be in the order of 95–100% [20]. Modelling by Kronfeld et al. of compiled data from several studies using diets with added fat/oil demonstrated that fat digestibility is maximized between 100 and 150 g/kg DM and sustained to at least 230 g/kg DM in horses [20]. Thus, diets with up to 230 g fat/kg DM from added corn oil, peanut oil, tallow, and animal-vegetable fat blends appear to be tolerated by horses without negatively impacting digestion of other nutrients, although the upper limit has not been determined for all types of fats [20]. A practical upper limit of 600 mL per day of added vegetable oil for a 500 kg horse or a maximum of 20% dietary energy as fat has been suggested [21]. The amount fed in the present study (278 g/kg DM) was greater than current recommendations, but care was taken to gradually increase the fat in the diet over the feeding period, and there were no clinical adverse effects of feeding this large amount of fat/oil.

Furthermore, this study suggests that even this amount of dietary fat has little adverse effect on apparent digestibility of the other nutrients evaluated. In the studies compiled by Kronfeld et al. [20], the feeding of diets with up to 230 g fat/kg DM had no negative effects on the digestion of DM, NDF or crude protein This appears to hold true for many different types of oil, including corn oil, linseed oil and blends of other vegetable oils added to the concentrate portion of the diet [22,23,24]. However, high levels of soybean oil (15–37%; but not lower levels) may negatively influence crude fibre, NDF and ADF digestion [25,26,27]. A further study established an upper limit for soy oil at 0.7 g/kg BW per day to maintain fibre digestion [28]. The mechanism by which soybean oil, but not other oils, might suppress fibre digestion is unknown. High fat diets have the potential to reduce mineral absorption due to the formation of mineral soaps in the small intestine, but few studies have examined this in equids. There is evidence that fat supplementation did not reduce the absorption of calcium and magnesium at rates of up to 2 g/kg BW per day [29], levels that were only slightly lower than the amount of fat provided in the present study.

Cereal grains are common feedstuffs used in equine rations to cost effectively increase the energy density of the diet, with the most common grains fed being oats, maize, and barley [30]. Maize is higher in starch than oats and barley and is therefore more energy dense. Processing of maize is very important due to the limitations of the equine gastrointestinal system, where digestion of grains ideally should occur in the small intestine by the action of enzymes and (to a lesser extent) microbes. The presence of undigested starch in the caecum, where it can undergo microbial fermentation, can result in hindgut acidosis, hindgut dysfunction, colic, and laminitis [31]. Starch digestion in the small intestine can be influenced by both horse factors and feed factors. First, the ability of α-amylase to digest starch is finite and will depend on both the amount secreted by the pancreas and activity within the intestinal lumen, as well as gastrointestinal transit time, with significant variability observed between individual horses [32,33]. Second, amylase needs to be able to access the starch granules contained within feed and the type of starch (e.g., ratio of amylose and amylopectin) will also influence digestibility [33,34,35]. Micronised maize was selected as the preferred source of grain for the present study, whereby the process of micronisation uses high temperatures for a short period of time to alter the starch structure, allowing greater availability of starch for digestion [35]. Further processing of grains aims to increase the surface area, allowing greater exposure of the starch granules to digestive enzymes [34,35]. Meyer et al. recommended a maximum of 2 g starch/kg BW per meal to prevent hindgut dysfunction [30]. The diets within the study reached a maximum of approximately 1.3 g starch/kg BW per meal, although it is recognised that recommended upper limits of starch intake to maintain general digestive health in equids remain controversial and is likely to depend on the context.

Starch digestion was approximately 80% in the control and fat-rich diet groups (although close to 100% in the cereal-rich diet group), which was somewhat surprising since it would be expected that any starch escaping digestion in the small intestine would be subjected to microbial fermentation in the large intestine [35]. Starch levels were very low in the fat-rich complimentary meals, and also relatively low in the control complimentary meals, whereas most starch digestibility studies have been conducted on animals adapted to feeds containing much higher levels of starch. Furthermore, all starch present in the fat-rich and control diets was provided by soya bean hulls, chaff and hay, and some of this starch may have been either non-fermentable or unavailable to the hindgut microbes. Additionally, although soya hulls contain relatively little starch, they are high in pectins and other soluble fibres [36], so it is conceivable that these might influence starch digestion [37]. The very low variation in apparent digestibility of starch observed in the cereal-rich diet group was because values were very high and approaching the asymptotic limit of 100%.

Animals consuming the cereal-rich and fat-rich diets both showed significantly greater apparent digestibility of DM and NDF compared with those fed the control diet. This was not necessarily expected in the grain-fed cohort, because it has been found that a single meal of unprocessed barley was sufficient to decrease caecal pH to the extent that the fibre digestibility of the diet was reduced [38]. However, providing pelleted or steam-flaked barley instead of ground barley appeared to limit the negative impact of starch on fibre digestibility [35,39], and gradually increasing the amount of grain fed will have facilitated adaptation, enabling more pre-caecal starch digestion. Protein levels in the two high-energy diets were similar, and also greater than the control diet, and this may have been a factor contributing to the improved digestibility of NDF [40].

Pelleted soya hulls were used as a carrier for the vitamin and mineral supplement powder and grain or fat/oil. Soya hulls are low in starch and other NSC, palatable and readily digested in the equine hindgut [41]. Two recent papers have specifically examined the effect of soya hulls on apparent nutrient digestibility in horses. Kabe et al. found that the inclusion of soybean hulls (up to 28% of a maize-based concentrate feed) had no effect on nutrient digestibility, ratios of short chain fatty acids, or lactate-producing Gram-positive bacteria in the faeces of crossbred mares [42]. Borghi et al. included soya hulls up to 40% of a concentrate feed and observed no major changes in the glycaemic effect or apparent nutrient digestibility [43]. In the present study, the inclusion of soya hulls was 28% and 37% in the cereal-rich and fat-rich complementary meals, respectively, and 49% of the control feed (although the total amount fed of the control diet was less than in the hypercaloric diets). Animals on the hypercaloric diets showed significantly greater apparent digestibility of gross energy and crude protein, which may have been a consequence of the calories rather than other factors. However, the increased amount of protein-rich soya hulls and lucerne chaff in those diet groups might have contributed also, because increased dietary protein levels have been suggested to improve the digestibility of crude protein, NDF and ADF in sport horses [40].

There were no significant differences in the digestibility of nutrients between breeds, which suggests that differences in digestibility might not be a major contributor to differences in metabolic phenotype among these breeds. The number of animals from the same breed in each diet group was small, and the observed variability was larger than predicted, so it cannot be discounted that the study was underpowered in that regard. However, visual inspection of Figure 1 did not suggest any breed-related trends that might become apparent with larger group sizes. Previous work comparing Icelandic horses with Danish warmbloods found higher digestibility of starch and crude fibre in Icelandic horses, although sample sizes were small, and age may have been a confounding factor [10]. Icelandic horses, like ponies, are considered ‘easy-keepers’. One hypothesis suggested by the authors was that maintenance energy requirements per unit of metabolic size might differ between horses and ponies, which might explain differences in body weight gain and body condition. Differences in feed retention times might also be a factor (which is difficult to assess). Several pony breeds were included in the present study, although they were all considered to be of similar ‘type’ and did not include Shetland ponies or miniature ponies, which might exhibit different metabolic profiles compared to other pony breeds. The ponies in this study did not demonstrate any greater heterogeneity among apparent digestibility variables compared with the Standardbred and Andalusian horses; however, our findings might not be generalisable to all pony breeds.

Higher mean body weight values were observed in the control group, despite this group exhibiting lower body condition, cresty neck score and total body fat mass [11]. Animals were not matched for weight prior to group allocation, as they were only blocked by breed prior to random group allocation, so this observation could be related to some taller or larger framed animals (across all breeds) being included in the control group. Interestingly, animals in the control group gained around 8% body weight over the 20-week feeding period [11]. Given that ad libitum hay was provided, when DM intakes were quantified it was calculated that digestible energy intakes were slightly above maintenance energy requirements [12]. However, since there were no changes in total body fat mass over the 20-week feeding period, weight gain was attributed to an increase in lean body mass, possibly due to the provision of complimentary meals containing a quality protein and fibre source, as discussed in the published longitudinal diet study [11].

One limitation of this study is the inability to differentiate differences in digestibility within the anatomical regions of the digestive tract, as we measured apparent total tract digestibility through the collection of faeces. Possible differences between breeds may include differences in pre-caecal digestibility or varying populations and numbers of microorganisms within the caecum and/or large colon. In a study by Dougal et al., a significant difference was observed in 16S rRNA gene terminal restriction fragment length polymorphism (T-RFLP) profiles comparing Thoroughbred horses with British native-breed ponies [44]. Furthermore, supplementing forage-based diets with starch or oil-rich complementary feeds has been associated with many differences in the faecal bacterial community compared with forage alone [45]. Therefore, the provision of additional energy (and perhaps other dietary components) to the hindgut microbes may well improve digestibility by improving the nutrient supply to the caecum and large intestine.

A recent report by Langner et al., comparing the faecal microbiome of Shetland ponies with warmblood horses during a weight gain study feeding 200% of metabolisable energy requirements (similar to the present study but extended over 2 years), described increases in Firmicute bacteria in the ponies, while horses exhibited decreases in the fibrolytic phylum Fibrobacteres, and both breeds showed an increase in the phylum Actinobacteria [46]. Due to the potential interplay between the intestinal microbiota and metabolic characteristics of an animal, such changes may well influence changes in insulin sensitivity and weight gain observed between breeds. However, based on the results of the present study, it appears that any differences are unlikely to result in overall functional differences in apparent digestibility.

The 24-h faecal collection period may also be a limitation of the present study, as most studies of apparent total tract digestibility in equids have typically collected faeces for 3 to 6 days. While a recent study recommended a 6-day faecal collection period following a 14-day dietary adaptation period, they also found that while the variability among digestibility estimates was improved when based on cumulative faecal collections, calculations based on 24-h faecal collections did not appear to differ significantly from one another [47]. Due to the nature of the study design and the large number of animals involved, a 24-h collection period was chosen for logistical reasons, although it is acknowledged that a longer collection period may have yielded different results. The aim of this study was not to precisely quantify digestibility values for each variable, but to compare digestibility among a well-characterised cohort of animals that included different breeds on equivalent terms, and shorter-than-recommended periods of total faecal collection are not without precedent [18]. Animals had been adapted to their respective diets and husbandry procedures for 20 weeks prior to carrying out faecal collections, and we considered the short collection period adopted to be sufficiently representative. Weather conditions were mild, and horses were well accustomed to being in individual side-by-side pens for prolonged periods of time, so stress was minimal. Another hypothesis for differences in weight gain and body condition between breeds may be the amount of free exercise they undertake within the paddock. We do not believe this was significantly different between breeds, although this influence cannot yet be excluded.

Since no breed differences were detected in apparent digestibility, bearing in mind the acknowledged study limitations, the question remains of why particular breeds appear more susceptible to being overweight. While differences in appetite and free choice exercise cannot be excluded on the basis of this study, the most likely explanation for differences in weight gain between the breeds involved in the study is related to differences in metabolic efficiency, including insulin secretion [3,48]. Ponies and Andalusian horses are known to release more insulin in response to a similar dietary intake of NSC (either simple sugars, fructans or starch) compared with Standardbreds; and are more prone to tissue insulin resistance, which further promotes hyperinsulinaemia [3,48]. Although these animals will also become obese on a high fat, low glycaemic load diet, the storage of fat and deposition of adipose tissue on a high glycaemic diet may be further promoted by the adipogenic effects of insulin [11].

## 5. Conclusions

This study found that horses and ponies had similar nutrient digestibility when adapted to the same diets and management conditions. The tendency towards increased adiposity in ‘easy keeper’ breeds such as ponies and Andalusian-type horse breeds may be more likely to reflect differences in metabolism, rather than differences in feed digestibility.

## Figures and Tables

**Table 1:** Proximate analysis and ingredient composition of the study diets [11]. Hay was sourced from a single batch for the duration of the study and provided ad libitum. Animals were fed either control, cereal-rich or fat-rich complementary feeds divided into two daily meals.

		Complementary Feed		
	Hay	Control	Cereal-Rich	Fat-Rich
DE (MJ/kg feed, DM basis)	7.1	9.4	12.4	16.4
DE (as fed; MJ/100 kg BW)		3.8	13.1	13.1
Nutrient (%; DM basis)				
CP	7.7	11.9	15.6	14.7
ADF	46.0	37.9	22.1	27.3
NDF	75.8	58.6	33.1	38.7
NSC	9.2	18.4	35.9	5.9
WSC	7.3	11.4	5.3	5.5
Starch	1.8	7.0	30.6	0.4
Fat	1.8	3.8	4.0	27.8
Ash	5.5	5.7	5.0	5.9
Complementary feed ingredients (g/100 kg BW)				
Soya hull pellets		200	300	300
Chaff		200	300	300
Micronised maize		0	455	0
Fat supplement		0	0	200
Vitamin/mineral supplement		6	6	6

ADF, acid detergent fibre; BW, body weight; CP, crude protein; DE, digestible energy; DM, dry matter; NDF, neutral detergent fibre; NSC, non-structural carbohydrate; WSC, water soluble carbohydrate.

**Table 2:** Morphometric characteristics of Standardbred horses, ponies and Andalusian horses adapted to consuming a control diet (*n* = 3 of each breed), cereal-rich diet (*n* = 4 of each breed) or fat-rich diet (*n* = 4 of each breed) at the time of digestibility collections (mean ± standard error).

	Standardbreds	Ponies	Andalusians
Body weight (kg)			
Control diet	489 ± 5	366 ± 32	515 ± 24
Cereal-rich diet	516 ± 22	345 ± 21	583 ± 29
Fat-rich diet	524 ± 11	321 ± 41	497 ± 34
Height at withers (cm)			
Control diet	155 ± 2	136 ± 2	153 ± 3
Cereal-rich diet	155 ± 3	131 ± 3	158 ± 5
Fat-rich diet	156 ± 2	131 ± 6	150 ± 6
Body conditon score (1–9 scale) *			
Control diet	5.3 ± 0.4	5.5 ± 0.5	5.8 ± 0.5
Cereal-rich diet	7.7 ± 0.2	8.0 ± 0.2	7.9 ± 0.2
Fat-rich diet	7.3 ± 0.2	7.5 ± 0.2	7.4 ± 0.2
Cresty neck score (0–5 scale) *			
Control diet	2.0 ± 0.3	2.3 ± 0.8	2.2 ± 0.3
Cereal-rich diet	3.4 ± 0.2	3.6 ± 0.2	3.9 ± 0.2
Fat-rich diet	2.9 ± 0.3	3.3 ± 0.4	3.5 ± 0.4

The statistical analysis to compare morphometric characteristics between groups has been previously reported [11]. No effects of breed or diet × breed were detected for any variables (all *p* > 0.05). * Significant difference between diet groups for these variables, with cereal-rich and fat-rich diet groups having significantly higher values than the control diet group (all *p* < 0.001).

**Table 3:** Measures of feed intake and faecal output during the 24-h total faecal collection period (mean ± standard error).

	Control Diet	Cereal-Rich Diet	Fat-Rich Diet
Feed DM intake (% BW)			
Hay	1.97 ± 0.10	1.73 ± 0.04	1.68 ± 0.08
Total (hay plus meals)	2.22 ± 0.08 ^a^	2.54 ± 0.04 ^b^	2.21 ± 0.08 ^a^
Feed DE intake (MJ/100 kg BW)			
Hay	14.0 ± 0.7	12.3 ± 0.3	11.9 ± 0.6
Total (hay plus meals)	17.8 ± 0.7 ^a^	25.4 ± 0.3 ^b^	25.0 ± 0.6 ^b^
Faecal DM output (% BW)	1.06 ± 0.07 ^a^	0.85 ± 0.09 ^a,b^	0.78 ± 0.07 ^b^
Faecal DM content (%)	20.2 ± 0.6	23.5 ± 1.8	23.1 ± 1.8

BW, body weight; DE, digestible energy; DM, dry matter; MJ, megajoules. ^a,b^ Different superscript letters indicate significant difference between diet groups (*p* < 0.05). No effect of breed was detected for any of these variables (all *p* > 0.05).

## Data Availability

The data presented in this study are available in the article and supplementary materials.

## Supplementary Materials

The following supporting information can be downloaded at: https://www.mdpi.com/article/10.3390/vetsci9010015/s1, Table S1: Apparent total tract digestibility of nutrients (%) in Standardbred horses, ponies and Andalusian horses adapted to eating a fibre-based control diet (n = 3 of each breed), hypercaloric cereal-rich diet (n = 4 of each breed) or hypercaloric fat-rich diet (n = 4 of each breed) over 20 weeks (mean ± standard deviation).
