# Supplementary material for: Comparison of Feed Digestibility between Ponies, Standardbreds and Andalusian Horses Fed Three Different Diets
Source: Vet Sci. 2021 Dec 31;9(1):15. doi: 10.3390/vetsci9010015 (PMC8778529; doi:10.3390/vetsci9010015)
Supplement: Supplementary file 1 [file vetsci-09-00015-s001.zip › vetsci-1494332-supplementary.pdf]

Supplementary Table S1. Apparent total tract digestibility of nutrients (%) in Standardbred horses, ponies and Andalusian horses adapted to eating a fibre-based control diet (n = 3 of each breed), hypercaloric cereal-rich diet (n = 4 of each breed) or hypercaloric fat-rich diet (n = 4 of each breed) over 20 weeks (mean ± standard deviation).

	Control	Cereal-rich	Fat-rich	Statistical analysis *	
Gross energy					
All breeds combined	50.5 ± 8.2 [a]	69.8 ± 10.6 [b]	66.1 ± 11.6 [b]	Diet	$p = 0.001$
Standardbreds	46.6 ± 7.4	75.9 ± 14.1	67.3 ± 18.7	Breed	$p = 0.73$
Ponies	48.8 ± 9.5	65.8 ± 10.2	65.3 ± 10.3	Diet × Breed	$p = 0.64$
Andalusians	56.2 ± 6.7	67.6 ± 5.8	65.8 ± 5.5		
Dry matter					
All breeds combined	52.5 ± 7.1 [a]	66.2 ± 5.3 [b]	63.9 ± 11.4 [b]	Diet	$p < 0.001$
Standardbreds	49.3 ± 3.2	60.7 ± 16.3	58.3 ± 15.9	Breed	$p = 0.64$
Ponies	50.3 ± 8.3	69.1 ± 9.8	66.8 ± 11.5	Diet × Breed	$p = 0.81$
Andalusians	57.8 ± 5.7	68.8 ± 5.4	66.8 ± 5.3		
Neutral detergent fibre					
All breeds combined	43.6 ± 7.5 [a]	63.5 ± 12.1 [b]	61.6 ± 11.1 [b]	Diet	$p < 0.001$
Standardbreds	40.4 ± 3.7	70.9 ± 12.5	61.7 ± 16.9	Breed	$p = 0.84$
Ponies	41.2 ± 8.1	60.2 ± 14.2	63.2 ± 10.4	Diet × Breed	$p = 0.49$
Andalusians	49.1 ± 8.8	59.4 ± 7.8	59.9 ± 6.8		
Starch					
All breeds combined	79.7 ± 10.3 [a]	97.1 ± 2.2 [b]	85.0 ± 11.4 [a]	Diet	$p < 0.001$
Standardbreds	77.0 ± 13.1	98.5 ± 1.2	85.4 ± 12.9	Breed	$p = 0.95$
Ponies	78.5 ± 12.0	94.4 ± 1.7	87.2 ± 10.8	Diet × Breed	$p = 0.79$
Andalusians	83.4 ± 8.4	98.1 ± 0.5	82.3 ± 6.7		
Crude protein					
All breeds combined	41.0 ± 10.9 [a]	61.3 ± 16.0 [b]	61.8 ± 12.2 [b]	Diet	$p = 0.004$
Standardbreds	40.6 ± 4.5	67.7 ± 25.9	64.6 ± 20.0	Breed	$p = 0.55$
Ponies	35.3 ± 14.8	56.2 ± 11.7	61.5 ± 11.1	Diet × Breed	$p = 0.84$
Andalusians	47.0 ± 11.5	60.1 ± 6.4	59.3 ± 0.6		
Crude fat					
All breeds combined	23.6 ± 13.5 [a]	55.3 ± 16.9 [b]	77.2 ± 12.6 [c]	Diet	$p < 0.001$
Standardbreds	18.3 ± 9.7	66.0 ± 24.8	80.8 ± 16.5	Breed	$p = 0.41$
Ponies	25.7 ± 14.5	50.0 ± 9.3	70.7 ± 13.6	Diet × Breed	$p = 0.64$
Andalusians	26.7 ± 19.1	54.9 ± 7.0	80.1 ± 6.3		

[a,b,c] Different superscript letters indicate significant difference between diet groups within the same row ($p < 0.05$).
* The statistical analysis was performed using a general linear model to evaluate the effects of diet, breed, and the interaction of diet × breed, with Bonferroni's *post hoc* test performed for pairwise comparisons.
